# Reduction of hexavalent chromium by *Ochrobactrum intermedium* BCR400 isolated from a chromium-contaminated soil

**DOI:** 10.1007/s13205-011-0038-0

**Published:** 2011-11-29

**Authors:** B. Kavita, Haresh Keharia

**Affiliations:** BRD School of Biosciences, Sardar Patel University, Vadtal Road, Vallabh Vidyanagar, 388120 Gujarat India

**Keywords:** Cr(VI) reduction, Biolog, Redox mediators, Chromate reductase, Quinone reductase

## Abstract

Hexavalent chromium-resistant *Ochrobactrum intermedium* BCR400 was isolated from chromium contaminated soil collected from Vadodara, Gujarat. It reduced 100 mg Cr(VI)/L completely in 52 h with initial Cr(VI) reduction rate of 1.98 mg/L/h. The Cr(VI) reduction rate decreased with increase in Cr(VI) concentration from 100 to 500 mg/L. The addition of anthraquinone-2-sulphonic acid (AQS) to culture *O. intermedium* BCR400 significantly enhanced its chromium reduction rate. The activation energy of AQS-mediated Cr(VI) reduction (120.69 KJ/mol) was 1.1-fold lower than non-mediated Cr(VI) reduction. An increase in the activities of quinone reductase and chromate reductase in cells grown in presence of AQS/AQS + Cr(VI) suggests their role in reduction of Cr(VI) by *O. intermedium*. Both chromate reductase and quinone reductase activities were FAD independent, required NADH as reductant, displayed maximum activity at pH (7.0) and temperature (30 °C). Thus Cr(VI) bioremediation potential of *O. intermedium* can be enhanced by augmentation of system with AQS as redox mediator.

## Introduction

Chromium (Cr) is one of the most widely used metals in variety of industrial processes such as steel production, dye manufacturing, wood preservation, chrome plating and leather tanning (Agarwal et al. 2008). Industrial effluents containing chromium compounds without proper treatment released directly or indirectly into natural water resources represent the major anthropogenic sources of Cr contamination of pristine environments. Chromium mainly exists as two stable oxidation states, Cr(VI) and Cr(III), other oxidation states of Cr viz., +V, +IV and +II are less stable and thus insignificant. Cr(VI) and Cr(III) differ completely in their physiochemical properties and toxicity (Ishibashi et al. 1990). Cr(VI) being highly toxic, mutagenic and carcinogenic (Costa 1997; Nishioka 1975; Wang et al. 1990) has been listed as one of the 17 chemicals posing greatest threat to humans by United States Environmental Protection Agency (USEPA) (McCullough et al. 1999). In-vivo, Cr(VI) due to the oxidizing activity results in generation of reactive oxygen species (ROS), which in turn causes oxidative damage to DNA, proteins and lipids (Cervantes et al. 2001). In contrast, Cr(III) being sparingly soluble, is also oxidatively more stable and thus less toxic in comparison to Cr(VI) (Dinakarpandian et al. 2004). Thus, biotransformation of Cr(VI) to the less toxic Cr(III) is considered as a feasible strategy for the remediation of Cr(VI) pollution worldwide (Diaz et al. 2008; Williams and Silver 1984). Effective physico-chemical methods have been developed for reduction of Cr(VI) to Cr(III), however, they suffer from limitations of use of chemicals and sludge generation with subsequent disposal problems. Biological processes on other hand are considered eco-friendly and less expensive (Losi et al. 1994; Saleh et al. 1989; Shannon and Unterman 1993). Several reports on microbial biotransformation of Cr(VI) to less toxic Cr(III) through direct enzymatic reaction or indirectly through metabolites exist in literature (Saleh et al. 1989; Puzon et al. 2002). The enzymatic chromate reaction occurs both in anaerobic as well as aerobic conditions (Cervantes et al. 2001). The anaerobic chromate reduction occurs in presence of membrane bound enzymes (Diaz et al. 2008). The Cr(VI) reducing activity in *Escherichia coli, Shewanella putrefaciens* and *Enterobacter cloacae* strains grown under anaerobic condition have been found to be located in membrane preparation (Myers et al. 2000; Shen and Wang 1993; Wang et al. 1990). In contrast, chromate reductases have been localized as soluble cytosolic proteins in most aerobic chromate reducing bacteria (Garbisu et al. 1998; Ishibashi et al. 1990; Puzon et al. 2002). Several oxidoreductases with unrelated metabolic functions have also been reported to catalyse Cr(VI) reduction in bacteria. Examples of such enzymes include, quinone reductases, iron reductases, nitro-reductases, flavin reductases and several NADH/NAD(P)H-dependent reductases of unknown function (Clark 1994; Gonzalez et al. 2003, 2005; Kwak et al. 2003; Mazoch et al. 2004; Suzuki et al. 1992). Gonzalez et al. (2005) demonstrated that the primary function of *E. coli* chromate reductase (*chr* R) was quinone reduction rather than chromate reduction.

Most of the studies reported on Cr(VI) reduction have concentrated on isolation, characterization and application of Cr(VI) reducing bacteria. Rehman et al. (2008) reported *Bacillus* sp. ev3 which was found to reduce 91% of 100 mg Cr(VI)/L after 96 h in growth medium. He et al. (2009**)** isolated *Ochrobactrum* sp. CsCr-3 which was found to reduce 100 mg Cr(VI)/L. Since bioremediation strategy of Cr(VI) involves its reduction to Cr(III), it would be apt to employ redox mediators to accelerate the process of electron transfer to Cr(VI). Recently, Zee and Cervantes (2009) have reviewed the impact of several quinone and viologen compounds (known to act as redox mediators) on redox biotransformation of contaminants. The redox mediators have been shown to accelerate the reductive degradation rate of several electron withdrawing recalcitrants while in certain instances such as Fe(III), U(VI), Tc(VII) they have even been required as prerequisites for initiation of their biodegradation (Fredrickson et al. 2006). Recently, Liu et al. (2010) reported that quinone redox mediator (lawsone) enhanced the rate of Cr(VI) reduction of by resting cells of *E. coli,* significantly.

This paper describes the studies on Cr(VI) reduction by *O. intermedium* BCR400, isolated from landfill site of chemical industry near Vadodara, Gujarat, India. Furthermore, we have investigated the role of anthraquinone 2-sulphonate (AQS) on reduction of Cr(VI) by *O. intermedium* BCR400 which has not been reported previously.

## Materials and methods

### Chemicals

Luria Bertani (LB) broth and redox mediators (namely, Anthraquinone sulphonate, Ethyl viologen, Benzyl viologen and Methyl viologen) were purchased from HiMedia Laboratories Ltd, Mumbai, India. Diphenyl carbazide and potassium dichromate (K_2_Cr_2_O_7_) were procured from Qualigens, India.

### Bacterial strain, identification and growth conditions

The bacterial strain, BCR400 tolerant to Cr(VI) was isolated from a long-term chromium polluted soil collected from landfill sites of Gorwa industrial zone (22°19′0″ North, 73°10′0″ East), Vadodara, Gujarat, India by enrichment culture technique. The culture was grown on LB agar plates (containing; Tryptone 10 g/L, Yeast Extract 5 g/L, NaCl 10 g/L) amended with 100 mg Cr(VI)/L. The K_2_Cr_2_O_7_ was used as source of Cr(VI) in all experiments.

BCR400 was identified using MicroLog 3 bacterial identification system employing GN2 and GEN III plates following the procedure recommended by the manufacturer (Biolog Inc., USA). Additionally, nucleotide sequence of 16S rRNA gene from BCR400 was also determined. The analysis of the nucleotide sequence was done using Blast-n tool at NCBI (http://blast.ncbi.nlm.nih.gov/Blast.cgi). The phylogenetic tree was constructed by the neighbour-joining method using MEGA version 4.0 (Tamura et al. 2007).

### Cr(VI) reduction by isolate *O. intermedium* BCR400

The 250 mL Erlenmeyer flasks containing 100 mL LB broth amended with Cr(VI) (100–500 mg/L) were inoculated with overnight grown cells of *O. intermedium* (*A*_660 nm _≈ 1.0). Uninoculated controls were used to compare abiotic Cr(VI) reduction during experiment. The inoculated cultures along with un-inoculated controls were incubated at 37 °C with shaking (150 rpm on orbital shaker) and 1 mL samples were withdrawn at regular time intervals to monitor growth and Cr(VI) reduction.

### Mediated Cr(VI) reduction by *O. intermedium* BCR400 in batch mode

LB broth amended with Cr(VI) (100 mg/L) was supplemented with redox mediators; AQS, ethyl viologen, methyl viologen and benzyl viologen to a final concentration of 1 mM. The Cr(VI) reduction was initiated after inoculation of culture flasks with *O. intermedium* BCR400. The Cr(VI) reduction as well as growth was monitored from samples withdrawn at different time intervals**.**

Control experiments were performed in the same manner except that no redox mediator was added to Luria Bertani broth.

Mediated Cr(VI) reduction by *O. intermedium* BCR400 in presence of different concentration of AQS was studied wherein, Luria Bertani broth amended with Cr(VI) (100 mg/L) was supplemented with AQS in the concentration range 0–5 mM. Cr(VI) reduction as well as growth was monitored from samples withdrawn at different time intervals.

### Effect of temperature on AQS-mediated Cr(VI) reduction

The AQS-mediated and non-mediated Cr(VI) reduction was determined at various incubation temperatures (25–40 °C). The Cr(VI) amended LB broth supplemented either with or without 1 mM AQS was inoculated with overnight grown culture of *O. intermedium* BCR400 and incubated at various temperatures (25–40 °C). The samples were withdrawn at regular time intervals to monitor residual Cr(VI).

The activation energy of the AQS-mediated and non-mediated Cr(VI) reduction by *O. intermedium* BCR400 was calculated by employing Arrhenius equation as follows:

1 $$ \ln k = - Ea/R \, T + \, \ln Ao $$

where *k* is the first-order rate constant (h^−1^), *Ea* is activation energy, *R* is the gas constant (KJ mol^−1^), *T* is the temperature (kelvin) and *Ao* is constant called the Frequency factor. Value of *E*a can be determined from the Slope (−*Ea*/*R*) of ln *k* versus 1/*T* plot (Santos et al. 2004).

### Preparation of cell-free lysate

*Ochrobactrum intermedium* BCR400 was grown in 200 mL Luria Bertani broth for 24 h at 30 °C. The cell pellet obtained upon centrifugation (8,603×*g* for 15 min) was resuspended in 3 mL of phosphate buffer (100 mM, pH 7.0). The resuspended cells were disrupted by sonication under ice bath (Sonics & Materials, Inc., USA) for 15 min (with 9 s on followed by 1 s off pulses) by supplying power at 35% amplitude. The resultant homogenate was centrifuged at 8,603×*g* for 30 min at 4 °C to remove cell debris and the clear supernatant was used as cell-free lysate for enzyme assays.

### pH and temperature optima of chromate and quinone reductase activities

The influence of pH and temperature on chromate and quinone reductase activity was assessed. For determination of optimal pH, the quinone and chromate reductase activity was assayed at 30 °C using 50 mM sodium acetate buffer (pH 5–5.5), potassium phosphate buffer (pH 6.0–7.5) and Tris–HCl buffer (7.5–8.5).

Optimal temperature was determined wherein the chromate and quinone reductase activity was assayed at various temperatures (ranging 20–50 °C) using thermostatic cuvette holder (ELICO DL 198 biospectrophotometer, Hyderabad, India)

## Analytical methods

### Enzyme assays

The NADH: quinone reductase and chromate reductase were assayed as described previously by Puzon et al. (2002). Briefly, quinone and chromate reductase activity was assayed spectrophotometrically at constant temperature of 30 °C by following the oxidation of NADH at 340 nm (Molar absorption coefficient 6.22 mM^−1^ cm^−1^). The reaction was initiated by addition of cell-free lysate to reaction mixture containing 50 mM phosphate buffer (pH 6.0) and 0.1 mM substrate (Lawsone as quinone reductase substrate and K_2_Cr_2_O_7_ as chromate reductase substrate).

One unit of enzyme activity was defined as the amount of enzyme required for oxidation of 1 μmole of NADH per min under standard assay conditions (Santos et al. 2004).

### Quantification of growth and Cr(VI)

Growth of *O. intermedium* BCR400 was monitored turbidometrically. The cell pellet obtained upon centrifugation of 1 mL of culture was resuspended in 1 mL distilled water and its absorbance was measured at 660 nm. The turbidometric measurements were then converted to dry biomass (g/L) using a correlation curve between absorbance of cell suspension (660 nm) and gravimetric biomass (g/L) measurement determined for *O. intermedium* BCR400 in our laboratory. The Cr(VI) concentration in the cell-free supernatant was measured using Diphenylcarbazide (DPC) reagent as described by Ishibashi et al. (1990). Briefly, the hexavalent chromium containing samples (in the range 1–10 μg) were acidified by adding 330 μL of 6 N sulphuric acid. To this acidified solution of the hexavalent chromium, DPC was added at concentration of 0.25% and final volume was made up to 10 mL. The mixture was incubated for 10 min at room temperature and the colour of DPC: Cr(VI) complex was measured by reading the absorbance at 540 nm.

### Protein estimation

The protein concentration of the cell-free extract (CFE) were estimated using Folin-phenol reagent by reading absorbance at 720 nm, following the principle of Lowry et al. (1951).

## Results and discussion

### Isolation and identification of BCR400

**Fig. 1:**
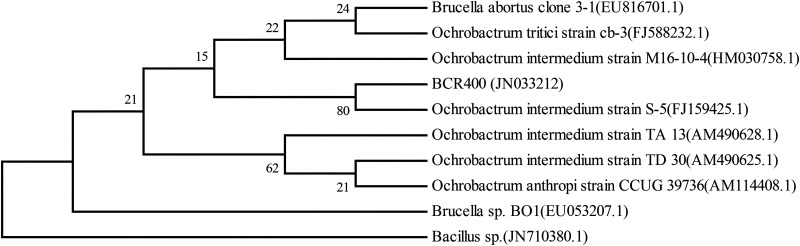
Phylogenetic affiliation based on 16S rRNA gene sequence comparisons over 1,406 nucleotides showing the relationship between members of family *Brucellaceae* and isolate BCR400. *The values at nod* represent percentage of 1,000 bootstrap replicates. *Numbers in bracket* represents GenBank accession numbers

A hexavalent chromium-reducing bacterial strain designated as BCR400 was isolated from the Cr(VI) contaminated soil collected from the landfill site of chemical industry in Gorwa GIDC of Vadodara, Gujarat, India. The isolate BCR400 was found to be motile, gram negative, short rod-shaped, possessed oxidase and catalase activities. On the basis of the carbon substrate utilization pattern employing GN2 as well as GENIII plates of BioLog, USA isolate BCR400 was identified as *O. intermedium* with similarity index of 0.74 and 99% probability. The identity of BCR400 was further confirmed by its 16S rRNA gene nucleotide sequence which also showed 99% identity to *O. intermedium.* (Accession number: JN033212). The phylogenetic relationship of the strain BCR400 with other related bacterial strains are presented in Fig. 1.

*Ochrobactrum intermedium* belongs to ά-proteobacteria subclass and representatives of this taxa have been isolated previously from chromium contaminated soils, by several researchers world-wide (He et al. 2009; Ozdemir et al. 2003; Sultan and Hasnain 2007; Thacker and Madamwar 2005). *O. intermedium* BCR400 exhibited growth up to 500 mg Cr(VI)/L. The optimal temperature and pH for growth was found to be 37 °C and pH 7.0, respectively (Data not shown).

### Time-course of Cr(VI) reduction by *O. intermedium* BCR400

**Fig. 2:**
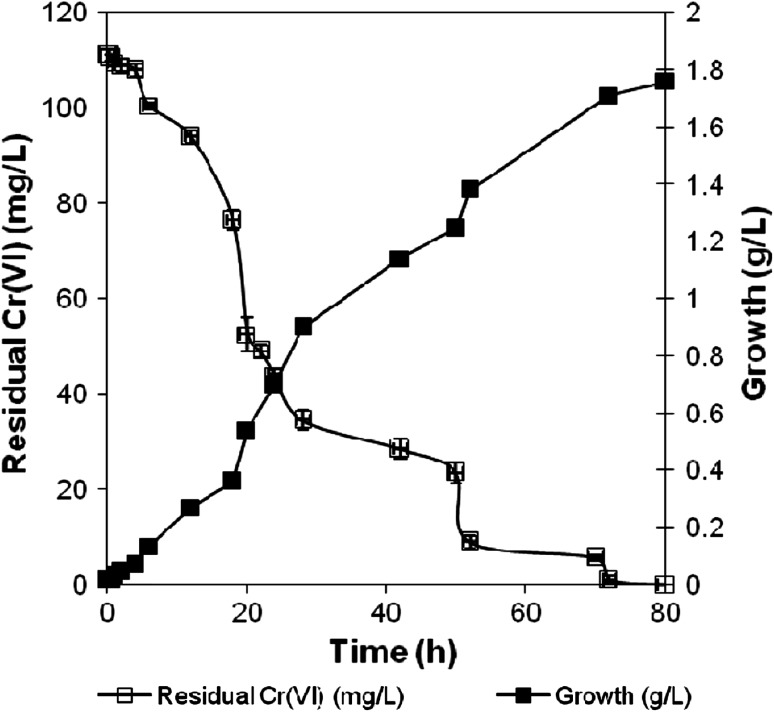
Cr(VI) reduction and growth profile of *O. intermedium* BCR400 in Luria Bertani broth amended with 100 mg Cr(VI)/L

Figure 2 shows that after a lag of 30 min growth and Cr(VI) reduction initiated simultaneously in an agitated batch culture of *O. intermedium* BCR400. The complete reduction of 112 mg Cr(VI)/L occurred within 72 h of incubation with initial reduction rate of 1.98 mg Cr(VI)/L/h. Thus, *O. intermedium* BCR400 not only showed resistance to Cr(VI) but also possessed ability to reduce Cr(VI), which is in agreement with report on *O. tritici* strain 5bvl1 by Branco et al. (2004). The resting cells of *O. intermedium* BCR400 did not show any significant reduction of Cr(VI). The growth associated Cr(VI) reduction suggests the role of actively metabolizing cells in Cr(VI) reduction.

### Effect of initial Cr(VI) concentration on growth and Cr(VI) reduction by *O. intermedium* BCR400

*Ochrobactrum intermedium* BCR400 was able to grow up to 500 mg Cr(VI)/L, while growth rate was found to decrease with increasing Cr(VI) concentration. The initial Cr(VI) reduction rate was found to increase up to 100 mg Cr(VI)/L without influencing the Cr(VI) reduction efficiency. However, further increase in Cr(VI) concentration up to 500 mg/L, negatively affected initial Cr(VI) reduction rate as well as extent of Cr(VI) reduction by *O. intermedium* BCR400 (Table 1). The decrease in amount of biomass produced with increasing Cr(VI) concentration may be attributed to consumption of increasing amount of reducing power of cell towards reductive detoxification of Cr(VI) (Branco et al. 2004; Megharaj et al. 2003). This means, electrons generated by oxidation of organic carbon sources which otherwise would be utilized for biosynthesis of cell components, seems to be diverted towards Cr(VI) reduction, thus slowing down growth (Sultan and Hasnain 2007). Similar observations on decrease in Cr(VI) reduction rate with increasing concentration of Cr(VI) have been reported by several researchers using different bacterial cultures (Garbisu et al. 1998; Megharaj et al. 2003; Sultan and Hasnain 2006).

**Table 1:** Effect on initial Cr(VI) concentration (50–500 mg/L) on Cr(VI) reduction and growth of *O. intermedium* BCR400

Initial Cr(VI) (mg/L)	Cr(VI) reduction (mg/L)	Initial Cr(VI) reduction rate (mg/L/h)	Maximum biomass (g/L)	Growth rate (g/L/h)
50	48.15 ± 2.61	1.29 ± 0.148	1.71 ± 0.001	0.0239 ± 0.002
100	99 ± 4.24	1.98 ± 0.034	1.38 ± 0.002	0.0206 ± 0.001
200	140 ± 8.48	1.20 ± 0.118	1.29 ± 0.001	0.0196 ± 0.001
300	131.5 ± 6.36	0.97 ± 0.002	0.93 ± 0.002	0.0132 ± 0.003
400	125 ± 5.65	0.844 ± 0.006	0.012 ± 0.0003	0.009 ± 0.0007
500	127 ± 0	0.730 ± 0.034	0.012 ± 0.0002	0.007 ± 0.0005

### Effect of redox mediators on the Cr(VI) reduction by *O. intermedium* BCR400

Inclusion of low molecular weight redox mediator along with the metal is emerging as general approach to enhance bacterial reduction of multivalent metal ions (Bond and Lovley 2002; Lovley et al. 1998, 1999). In the present study, AQS, ethyl viologen, methyl viologen and benzyl viologen at 1 mM concentration were included as redox mediator as they have been reported to act as potential redox mediator in the biotransformation of recalcitrant pollutants like sulphonated azo dye, iron, 2,4 dichlorophenoxy acetic acid and carbon tetra chloride (Bond and Lovley 2002; Ling et al 2009; Maithreepala and Doong 2009; Wang et al. 2009). *O. intermedium* BCR400 exhibited higher initial Cr(VI) reduction rate in presence of 1 mM AQS, ethyl and methyl viologen (Table 2). In presence of 1 mM AQS, *O. intermedium* BCR400 reduced Cr(VI) at 1.4-fold higher rate (2.88 mg Cr(VI)/L/h) than in absence of any redox mediators (1.98 mg Cr(VI)/L/h).

**Table 2:** Effect of redox mediators on Cr(VI) reduction and growth of *O. intermedium* BCR400

Redox mediators (1 mM)	Cr(VI) reduction (mg/L)	Initial Cr(VI) reduction rate (mg/L/h)	Biomass (g/L)	Initial growth rate (g/L/h)
None	99.5 ± 0.353	1.98 ± 0.034	1.01 ± 0.001	0.038 ± 0.0001
AQS	99.25 ± 1.06	2.9 ± 0.123	1.35 ± 0.010	0.052 ± 0.0021
Ethyl Viologen	102 ± 2.3	2.12 ± 0.062	0.825 ± 0.006	0.022 ± 0.0003
Methyl Viologen	103 ± 4.7	2.14 ± 0.048	0.768 ± 0.011	0.021 ± 0.0003
Benzyl Viologen	60.87 ± 6.9	0.85 ± 0.097	0.047 ± 0.003	0.0012 ± 0.00008

Anthraquinone 2-sulphonate has been reported as powerful mediator for reductive biotransformation of several organic recalcitrants (Liu et al. 2010; Zee and Cervantes 2009). Although ethyl and methyl viologen also enhanced Cr(VI) reduction rate, they were found to inhibit growth of *O. intermedium* BCR400 (Table 2). Benzyl viologen severely inhibited the growth of *O. intermedium* BCR400.

Quinones are known for their potential role as redox centres in humic acid and therefore the Cr(VI) reduction rates in presence of AQS may attributed to their electron shuttling ability between *O. intermedium* BCR400 and Cr(VI). Zee and Cervantes (2009) suggested that the oxidation reduction potential of any mediator should not be much lower than −0.320 V, which is the lowest oxidation reduction potential of cofactor (NADPH) in the cell otherwise it would not be reduced significantly. Hence, the transfer of electrons from AQS to Cr(VI) seems to be thermodynamically favourable phenomenon as the standard redox potential (*E*°) of AQS is −0.218 V, which is significantly lower than the redox potential of CrO_4_^2−^ (1.28 V) and higher than −0.320 V. According to the above hypothesis, the poor mediating ability of ethyl viologen and methyl viologen may be explained by their much lower oxidation reduction potential (−0.480 and −0.440 V, respectively) than −0.320 V.

### Effect of AQS concentration on Cr(VI) reduction

The optimum AQS:Cr(VI) ratio for Cr(VI) reduction by *O. intermedium* BCR400 was determined by varying the concentration of AQS while maintaining Cr(VI) concentration constant. It is clearly evident from Table 3, that initial Cr(VI) reduction rate of *O. intermedium* BCR400 increased with increase in AQS concentration from 0.1 to 1.0 mM. Further increase in AQS concentration to 2 mM caused decrease in initial Cr(VI) reduction rate as well as growth of *O. intermedium* BCR400. The lower Cr(VI) reduction rate at suboptimal AQS concentrations may be explained by distribution of electrons between AQS-mediated reduction and direct Cr(VI) reduction. On the other hand, higher AQS concentrations (2 mM) itself may be inhibitory to cell growth thus negatively influencing Cr(VI) reduction (Ling et al. 2009). This is because at higher AQS concentrations, the accumulation of reduced AQS (AQH_2_S), in absence or limiting concentration of suitable electron acceptor, would transfer electrons to O_2_, resulting in generation of ROS and thereby causing cell death (Sedlacek and Kucera 2010). The maximum reduction rate of Cr(VI) as well as growth of *O. intermedium* BCR400 at optimal AQS (1 mM) in medium containing 100 mg Cr(VI)/L may be due to efficient relay of electrons from primary electron donor to AQS to Cr(VI) mediated through action of reductases. Rau et al. (2002) also observed highest reduction of azo dye amaranth in presence of 1 mM AQS.

**Table 3:** Effect on AQS concentration (0–2.0 mM) on Cr(VI) reduction and growth of *O. intermedium* BCR400

AQS (mM)	Cr(VI) reduction (mg/L)	Initial Cr(VI) reduction rate (mg/L/h)	Biomass (g/L)	Initial growth rate (g/L/h)
0	99.5 ± 0.353	1.98 ± 0.0	1.01 ± 0.001	0.038 ± 0.0001
0.1	81.2 ± 3.46	1.49 ± 0.0618	0.993 ± 0.0127	0.029 ± 0.0020
0.2	96.85 ± 1.62	1.72 ± 0.0290	1.04 ± 0.0063	0.030 ± 0.0023
0.5	97.5 ± 0.707	2.2 ± 0.141	1.08 ± 0.0	0.031 ± 0.0026
0.75	98.8 ± 0.21	2.43 ± 0.0094	1.22 ± 0.003	0.035 ± 0.002
1.0	99.25 ± 1.06	2.9 ± 0.123	1.35 ± 0.010	0.052 ± 0.0021
1.5	99.02 ± 1.87	2.61 ± 0.022	1.26 ± 0.0013	0.037 ± 0.0011
2.0	83 ± 1.41	1.6 ± 0.141	0.939 ± 0.042	0.027 ± 0.0010

### Intracellular quinone and chromate reductase activities of *O. intermedium* BCR400 induced in the presence of AQS

The intracellular quinone and chromate reductase activities in *O. intermedium* BCR400 grown in LB were found to be 0.0063 ± 0.0010 and 0.0042 ± 0.0001 U/mg, respectively (Table 4). The quinone and chromate reductase activity was found to increase in *O. intermedium* BCR400 grown in presence of Cr(VI) (100 mg/L) or 1 mM AQS. Furthermore, the extent of quinone reductase induction was found to be higher in cells grown in presence of AQS than in presence of Cr(VI); whereas the extent of chromate reductase induction was not profoundly influenced by Cr(VI) over AQS. It is noteworthy to mention here that the presence of AQS + Cr(VI) synergistically influenced the level of intracellular chromate reductase and not quinone reductase. This suggests that Cr(VI) reduction in *O. intermedium* BCR400 may occur directly by chromate reductase as well as by reduced AQS (AQH_2_S) formed upon action of quinone reductase. Furthermore, both types of reductases seem to have relaxed substrate specificity; chromate reductase being more specific for Cr(VI), while quinone reductase being more specific for quinoid compounds (Puzon et al. 2002; Rau et al. 2002).

**Table 4:** Quinone and chromate reductase activities (U/mg) of *O. intermedium* BCR400 grown in Luria Bertani (LB) broth amended with either Cr(VI) or AQS or both Cr(VI) and AQS

Growth medium	Enzyme Activity (U/mg)	
	Quinone reductase	Chromate reductase
LB	0.0063 ± 0.0010	0.0042 ± 0.0001
LB + Cr(VI)	0.0139 ± 0.0028	0.013 ± 0.00018
LB + AQS	0.026 ± 0.004	0.016 ± 0.0005
LB + AQS + Cr(VI)	0.025 ± 0.0036	0.020 ± 0.00054

**Fig. 3:**
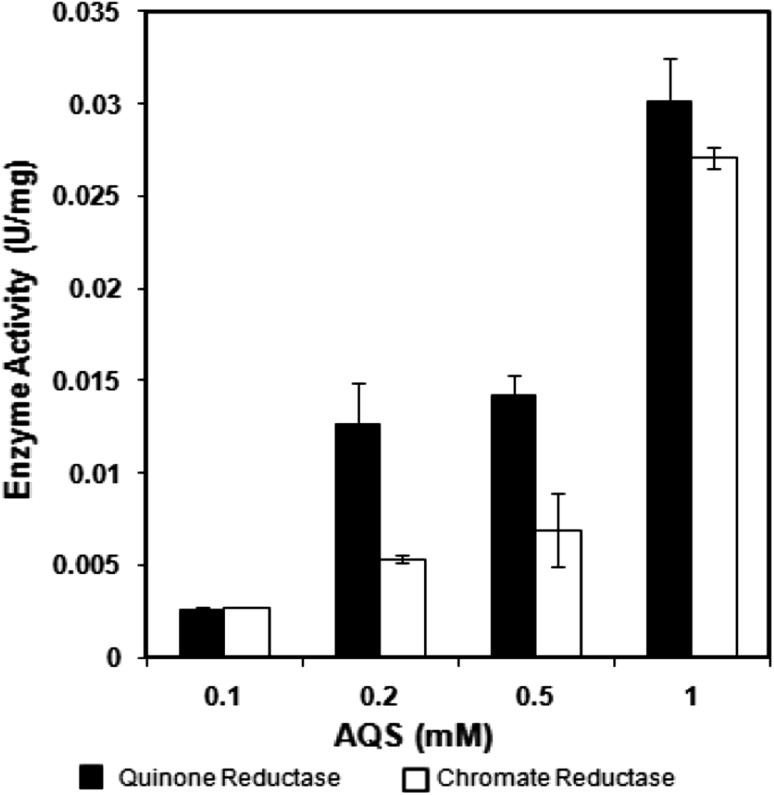
Quinone and chromate reductase activity (U/mg) of *O. intermedium* BCR400 grown in presence of increasing AQS concentrations (0–1.0 mM)

When *O. intermedium* BCR400 was grown in the presence of varying AQS concentration (0.1–1 mM) the intracellular quinone and chromate reductase activities were found to increase from 0.0025 ± 0.00019 to 0.030 ± 0.0024 U/mg protein and 0.0027 ± 0.00002 to 0.027 ± 0.0005 U/mg, respectively (Fig. 3). Furthermore, a strong and non-linear positive co-relation between the concentration of AQS and activities of both the enzymes (γ > +90) was observed. This suggests the role of AQS in induction of quinone and chromate reductases in *O. intermedium* BCR400.

**Fig. 4:**
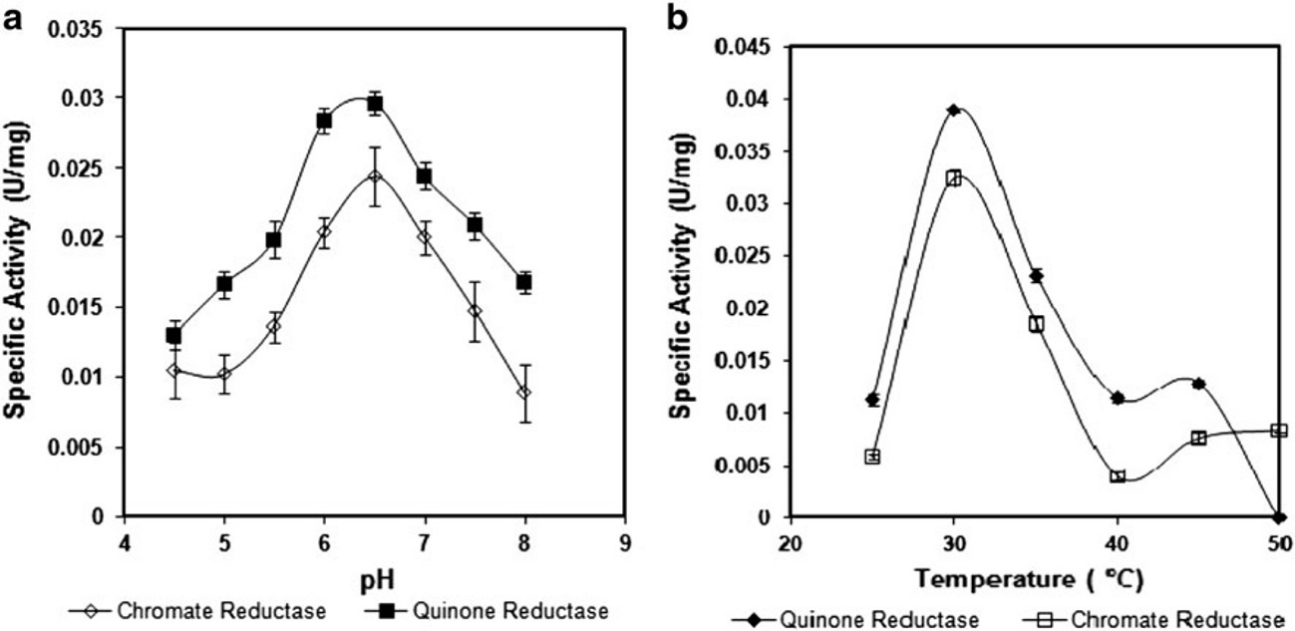
Effect of pH (**a**) and temperature (**b**) on quinone and chromate reductase activity (U/mg) of *O. intermedium* BCR400

Both quinone and chromate reductase were found to follow similar activity profile over a pH range 5.0–8.5 with maximum activities at pH 7.0. Also, the quinone and chromate reductase exhibited similar activity profile over a temperature range 25–45 °C, with minor variation at 50 °C (Fig. 4). It is not possible to comment on these activities unless they are purified and characterized independently, if at all they exist as different enzymes.

### Effect of temperature on AQS-mediated Cr(VI) reduction

Santos et al. (2004) observed that rate of Anthra Quinone Di-Sulphonate (AQDS)-mediated azo dye reduction was significantly higher in comparison to non-mediated azo dye reduction suggesting the role of AQDS in lowering the activation energy (*Ea*) of azo dye reduction. We monitored initial Cr(VI) reduction rate of *O. intermedium* BCR400 cells in presence and absence of 1 mM AQS at different temperatures (25–35 °C). The non-mediated Cr(VI) reduction rate of *O. intermedium* BCR400 increased from 0.005 to 0.02 h^−1^ with increase in temperature from 25 to 30 °C and remained constant thereafter with further increase in temperature up to 35 °C. Similarly, the mediated Cr(VI) reduction rate increased from 0.007 to 0.034 h^−1^ with increase in temperature from 25 to 35 °C (Table 5).

**Table 5:** Effect of temperature on Cr(VI) reduction rate (h^−1^) of *O. intermedium* BCR400 in presence (mediated) and absence (non-mediated) of 1 mM AQS

Temperature (°C)	Cr(VI) reduction rate (h^−1^)	
	Non-mediated	Mediated
25	0.005 ± 0.0001	0.007 ± 0.0015
30	0.020 ± 0.0001	0.022 ± 0.0012
35	0.021 ± 0.0002	0.034 ± 0.0011

Furthermore, the activation energy of AQS-mediated Cr(VI) reduction (120.69 KJ mol^−1^) was found to be 1.1-fold lower than non-mediated Cr(VI) reduction (133.86 KJ mol^−1^). Santos et al. (2004) observed that activation energy of AQS-mediated azo dye (reactive red 2) reduction (22.9 KJ mol^−1^) by anaerobic sludge was 1.2-fold lower than non mediated dye reduction (27.9 KJ mol^−1^) in anaerobic sludge.

There have been many studies on the role of redox mediators such as AQS, AQDS and Lawsone in bacterial azo dye reduction under anaerobic condition. Rau et al. (2002) proposed that mechanism for such redox mediator dependent reduction of azo dyes consist of two independent reaction steps: First, the quinones are enzymatically reduced to the corresponding hydroquinone (Ling et al. 2009; Rau and Stolz 2003) and second, the hydroquinones cleave the azo dyes in purely chemical reaction. Following the proposed mechanism by Rau et al. (2002), Ling et al. **(**2009**)** suggested that entire reaction rate depends on enzymatic reduction of redox mediators. Accordingly, the effectiveness of any mediator depends on the presence of membrane bound or intracellular reductase with mediator reducing ability.

Hence, a hypothetical model for AQS-mediated multi-step Cr(VI) reduction by *O. intermedium* BCR400 has been proposed. In the first step, AQS may be reduced to AQH_2_S (hydraquinone) by quinone reductase. In the second step, the AQH_2_S reduces Cr(VI) by 2e^−^ transfer, thereby reducing it to Cr(IV) in a purely redox chemical reaction. The direct reduction of Cr(VI) to Cr(IV) by AQH_2_S would as well prevent ROS generation and thereby exerting protective effect on the growth of *O. intermedium* BCR400. The further reduction of Cr(IV) to Cr(III) may be catalysed by single e-transfer catalyzed by specific chromate reductase or non-specific reductases. The model is based on several lines of evidences:

1. The supplementation of AQS to nutrient growth medium amended with Cr(VI), not only had protective effect against toxicity due to Cr(VI) but also enhanced the rate of Cr(VI) reduction by *O. intermedium* BCR400.
2. Increase in both quinone and chromate reductase activity (U/mg protein) was observed in cell-free lysate prepared from *O. intermedium* BCR400 grown in presence of AQS or Cr(VI) + AQS.
3. The CFE of *O. intermedium* BCR400 grown in presence variable AQS concentration (0–1mM) displayed high, non-linear positive correlation between the concentration of AQS and activities of both quinone reductase and chromate reductase (γ > +90).

## Conclusion

The *O. intermedium* BCR400 isolated from chromium-contaminated site reduced 100 mg Cr(VI)/L efficiently within 52 h. The augmentation of 1 mM AQS in the medium enhanced the Cr(VI) reduction efficiency of *O. intermedium* BCR400. The activation energy required for AQS mediated Cr(VI) reduction was found to be 1.1-fold lower than non-mediated Cr(VI) reduction by *O. intermedium* BCR400. Furthermore, AQS was found to induce both quinone and chromate reductase activities in cells of *O. intermedium* BCR400, which further exhibited similar activity profile over a pH range 4.5–8.0 and temperature range 25–45 °C. On the basis of results, it is proposed that AQS-mediated Cr(VI) reduction by *O. intermedium* BCR400 is a two step process, wherein the first step involves enzymatic reduction of AQS to AQH_2_S and in second step AQH_2_S reduces Cr(VI) to Cr(IV) in a purely chemical reaction. The Cr(IV) thus formed would be reduced to Cr(III) by cellular specific or non-specific enzymes. However, this hypothesis needs to be investigated using purified enzymes and sophisticated tools particularly required monitoring different valence forms of Cr produced during its reduction.
